# Longitudinal Study on Health-Related Quality of Life in a Cohort of 96 Patients with Common Variable Immune Deficiencies

**DOI:** 10.3389/fimmu.2014.00605

**Published:** 2014-11-26

**Authors:** Stefano Tabolli, Patrizia Giannantoni, Federica Pulvirenti, Fabiola La Marra, Guido Granata, Cinzia Milito, Isabella Quinti

**Affiliations:** ^1^Health Services Research Unit, Istituto Dermopatico dell’Immacolata (IDI), Istituto di Ricovero e Cura a Carattere Scientifico (IRCCS), Rome, Italy; ^2^Department of Molecular Medicine, Sapienza University of Roma, Rome, Italy

**Keywords:** common variable immune deficiencies, health-related quality of life, SF-36, GHQ-12, immunoglobulins

## Abstract

Health-related quality of life (HRQoL) in common variable immunodeficiency diseases (CVID) was evaluated by different tools, which were mainly used to compare different schedules of immunoglobulins administration in cross-sectional or short-term longitudinal studies. We assessed the HRQoL and psychological status of CVID patients in a longitudinal study over a 6-year period by a generic, non-disease-specific instrument (SF-36), and by a General Health Questionnaire (GHQ-12) for the risk of depression/anxiety. At baseline, 96 patients were enrolled. After 1 year, a second assessment was performed on 92 patients and, after 6 years, a third assessment was performed on 66 patients. Eighteen patients died during the study time. HRQoL was low, with mental health scales less affected than physical scales. A decline in the score on SF-36 scales was observed between the first and the third assessment for the Physical Functioning, Body Pain, General Health, Social Functioning, and Role-Emotional scales. The General Health scale showed a lower score in these patients, when compared to patients with other chronic diseases. Approximately one-third of the patients were at risk of anxiety/depression at all observation times, a percentage that reached two thirds of the patients, considering only the group of females. Over the 6 years of the study, the health condition of 11/66 patients worsened, passing from “GHQ-negative” to “GHQ-positive”; their score on SF-36 scales also decreased. A decrement of one point in each of the Physical Functioning, Vitality, Social Functioning, and Mental Health SF-36 scales increased the risk of developing anxiety/depression from three to five percent. A negative variation of the Physical Functioning score increased the risk of psychological distress. In a survival analysis with dichotomized variables, Physical Functioning scores <50 were associated with a relative risk (RR) of 4.4, whereas Social Functioning scores <37.5 were associated with a RR of 10.0. In our study, it was the clinical condition, as opposed to the different treatment strategies with immunoglobulins, which had a major role on the deterioration of HRQoL. Moreover, in a quality-of-life evaluation, disorders such as anxiety/depression should be assessed, as they yet often go unrecognized. Our results might be helpful in the interpretation of currently available data on quality of life in CVID patients.

## Introduction

The health-related quality of life (HRQoL) is a multidimensional concept that encompasses measurements of physical, psychological, and social well-being and assesses the individual’s perception of the impact of illness on his/her life (1).

Common variable immunodeficiency diseases (CVIDs) represent a heterogeneous group of rare chronic disorders of the immune system (2). The prognosis can vary from benign to very complex conditions (3). There is substantial evidence that the standard replacement treatment with immunoglobulins prolongs survival, reduces morbidity, and exerts a positive effect on the patients’ HRQoL (4). Until now, different tools to evaluate HRQoL in CVID were used mainly to assess the patients’ outcome and satisfaction related to different treatment choices (i.e., intravenous-IVIG vs. subcutaneous-SCIG immunoglobulin routes of administration) (5, 6). However, HRQoL in CVID should be assessed in the frame of the wide spectrum of the severity of the disease, taking into consideration the long life course of the disease.

There are several critical reasons to evaluate the available data on HRQoL in CVID. The absence of a disease-specific questionnaire is a major limitation. Only observational or short-term longitudinal studies on small cohorts were performed. Differences in HRQoL were mainly evaluated to compare different treatment regimens and routes of immunoglobulins administration.

With such limitations in evaluating quality of life in CVID, both ourselves and others (7–9) have used generic, non-disease-specific instruments, such as the Health Status Questionnaire (Medical Outcome Study 36-Items Short Form, SF-36) and the General Health Questionnaire (GHQ-12 Items) for the psychological assessment. All the studies agreed that CVID patients have a poor quality of life, especially in the physical domain, suffer a lot, and are at risk of psychological distress.

Because of the long lifetime of the disease, it is possible to speculate that quality of life in a population affected by a clinical and immunological heterogeneous disease may vary depending on age, treatment, clinical conditions, associated diseases, personal attitude, etc. Thus, it is evident that studies about the HRQoL outcome in CVID should be extended from a simple assessment to multimodal and longitudinal assessments and should include the patients’ reported outcome measures.

The aim of this study was to assess the HRQoL and psychological status of patients with CVID over a 6-year period, using SF-36 and GHQ-12 questionnaires. Moreover, we investigated whether the psychological problems of patients, such as risk of depression/anxiety, could be associated with their health status.

## Materials and Methods

### Study design

**Figure 1:**
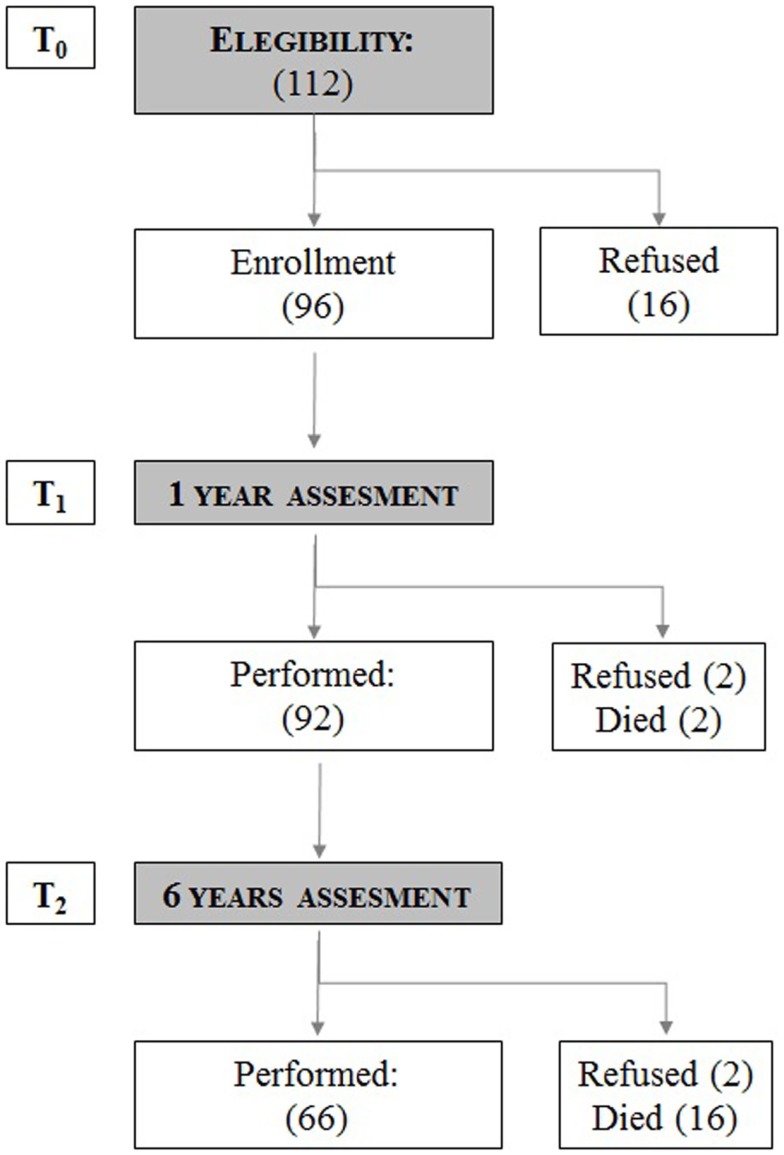
**Study flow chart**. The number of patients analyzed at baseline (T0), after 1 year (T1), and after 6 years (T2). The number of patients who refused to continue the study and the number of patients who died during the study time is shown.

For this observational, longitudinal, cohort study (Figure 1), performed in a day hospital setting, patients’ participation was obtained after signing an informed consent. The study was conducted in the period 2008–2013. One hundred twelve CVID patients were informed and considered eligible for the study. At basal time (T0), 96 patients were enrolled and 16 patients refused to participate. After 1 year, at the second assessment (T1) 92 out of 96 patients were evaluated (between T0 and T1 two patients died and two patients refused to continue the study). After 6 years, 66 out of 92 patients were available for the third assessment (T2). Between T1 and T2, 12 patients refused to participate and 16 patients died in the 5-year period. At the end of the study, data for the 66 patients were considered, taking into account all three observations. At T0, T1, and T2, patients were asked to fill in questionnaires concerning their health status and the possible presence of risk for depression and/or anxiety. The severity of disease judged by the physician (Physician Global Assessment, PhGA) and by the patient (Patient’s Global Assessment, PtGA) was recorded. The study protocol was approved by the Ethical Board of the Sapienza, University of Rome.

### Patients

We enrolled 112 patients with CVIDs attending our Reference Center for Primary Immune Deficiencies. Patients were diagnosed according to the ESID/PAGID criteria for CVIDs (10), based on IgG <500 mg/dL, IgA 2 SD below age-specific reference range, age onset >4 years, poor response to vaccines, and exclusion of other causes of hypogammaglobulinemia. No genetic causes of CVID were identified in this cohort. A detailed set of data was available, since all patients with a diagnosis of CVID have been regularly followed up in our center according to the Italian guidelines (www.aieop.org); their clinical and immunological data have been collected regularly in a national database, once a year. The dataset included age, date of CVID diagnosis, immunological data, including lymphocyte subsets and serum IgG, IgA, and IgM levels determined every 3 months, clinical manifestations, route, doses, and intervals of Ig replacement, and occurrence of adverse reactions. Route, dosage, and interval of Ig replacement were recorded once a month. At T0, 84 patients were on replacement therapy with IVIG and 12 with SCIG. Between T0 and T2, 6/66 patients shifted from IVIG to SCIG. High-resolution chest computerized tomography (HRCT) scans was performed once in every 4 years in all patients according to national guidelines. All patients have been on IVIG or SCIG replacement for at least 5 years.

### Questionnaires

We used validated tools: SF-36, GHQ-12, and PGA questionnaires.

#### SF-36

Despite the fact that it was designed as a generic health status indicator for use in population surveys and health policy evaluation studies, the SF-36 can also be used as an outcome measure (11, 12). The SF-36 includes 36 items in a Likert-type or forced-choice format, intended for measuring the following eight dimensions: physical functioning (*PF*, limitations in performing physical activities such as bathing or dressing), role-physical (*RP*, limitations in work and other daily activities as a consequence of physical health), bodily pain (*BP*, how severe and limiting pain is), general health (*GH*, how general personal health is perceived by the patient), vitality (*VT*, feeling tired and worn out vs. feeling energetic), social functioning (*SF*, interference with regular social activities because of physical or emotional problems), role-emotional (*RE*, limitations in work and other daily activities as a consequence of emotional problems), and mental health (*MH*, feeling nervous and depressed vs. peaceful, happy, and calm). Scores for each domain ranged from 0 to 100, with higher scores indicating better health. Two additional summary measures, the physical component summary (PCS) and mental component scores (MCS), cross-culturally validated in the framework of the International Quality of Life Assessment project for the Italian version of the SF-36, were also obtained.

#### GHQ-12

The GHQ-12 is a self-administered, 12-item questionnaire, designed to measure psychological distress and to detect current non-psychotic, psychiatric disorders, such as depression and anxiety (13, 14). Answers are given on a 4-point scale; for instance, the item “in the last weeks, did you feel under strain?” allows for the following answers: “no,” “not more than usual,” “more than usual,” and “much more than usual.” When scored with the binary method (0–0–1–1), the GHQ-12 can be used as a screening tool to detect minor non-psychotic, psychiatric disorders, yielding final scores that range from 0 to 12. Operationally, patients scoring 4 or more were considered as “GHQ-positive” (GHQ+).

#### PGA

For each patient, an overall clinical severity evaluation of the disease was given by the provider and by the patient him/herself. The PhGA and the PtGA consisted of the following questions respectively: “In your opinion, compared to other patients with the same condition, how severe is the disease of patient X?” and “In your experience, how severe is your disease?” Answers were given on a 5-point scale: “very mild,” “mild,” “moderate,” “severe,” and “very severe.” For the purpose of statistical analysis, “very mild”/“mild” were considered as low severity and “severe”/”very severe” as high severity, and were grouped. The same physician at T0, T1, and T2 recorded her evaluation at the end of the visit. Patients recorded his/her evaluation after the completion of the questionnaires.

### Statistics

In the first part, for descriptive analyses and comparisons among groups, we used *t*-test for independent samples and ANOVA for the comparison of mean values, due to the samples’ size. Chi-squared test was also used for the comparison of percentages. For the 66 patients present in each observation, paired tests were used. In the second part, we performed a logistic regression analysis to assess the independent role of SF-36 scales on GHQ-12 deterioration over time. In the last part, in order to investigate factors predicting mortality, survival analysis was performed, both through Kaplan–Meier curves and Cox regression analysis. All analyses were performed using the Stata version 11 (Stata Corp, College Station, TX, USA).

## Results

The study design flow chart on CVID patients enrolled in the cohort study is shown in Figure 1. One hundred twelve patients were enrolled; 96 patients accepted to participate in the study [M/F: 50/46; mean age: 48.2 ± 17.0 years old (range 14–85); mean time of disease since diagnosis: 10.7 years (range 5–36)]; 92 patients [M/F: 47/45; mean age: 49 ± 4.9 years old (range 15–86)] completed the second assessment. Sixty-six patients [M/F: 32/34; mean age: 50 ± 5.7 years old (range 20–76)] completed the T0, T1, and T2 assessments. Thirty patients refused to participate (16 at T0, 2 at T1, and 12 at T2). Eighteen patients [M/F: 9/9; mean age: 62.9 ± 14.7 years old (range 39–88)] died in the 6-year period. Causes of death were: gastrointestinal cancer (5 patients), lymphoproliferative diseases (5 patients), chronic lung disease (CLD) (2 patients), cirrhosis (1 patient), and granulomatosis (5 patients).

### Descriptive analyses

#### Patients’ characteristics at baseline and comparison between SF-36 in CVID and in other chronic diseases

At baseline (T0), the characteristics of patients were those reported in our study published in 2012 (7). A summary of our previous data on HRQoL is reported in Table 1. Being female, older, and affected by CLD and chronic diarrhea proved to be major risk factors leading to a poor quality of life. The basal mean scores for SF-36 scales were also compared to those reported (12) on patients affected by other chronic diseases (Table 2). HRQoL was lower than that reported in generally healthy population, with mental health scales less affected than physical scales. In CVID, better scores for PF, BP, VT, SF, RE, and MH scales were observed, while RP and GH scales showed a lower score in comparison to patients with all other disease entities, with the exception of patients affected by heart failures who showed the lowest scores. The different age range of patients in each group (older in cancer, younger in CVID) has a significant effect on profiles of SF-36 average scores. Therefore, one must be cautious in the interpretation of differences in SF-36 scores between pathologies: they cannot be entirely attributed to the “pure” effect of the disease. This consideration is even more valid in the longitudinal study.

**Table 1:** **SF-36 mean values (SD) and clinical characteristics of CVID patients**.

	*n*	PF	RP	BP	GH	VT	SF	RE	MH	PCS	MCS
All	96	72 (25)	47 (42)	67 (26)	39 (24)	55 (22)	69 (22)	68 (40)	66 (20)	40 (12)	43 (12)
**Gender**											
Male	50	78 (24)	59 (41)	73 (25)	44 (27)	62 (20)	72 (23)	74 (38)	69 (20)	43 (12)	45 (12)
Female	46	**66 (25)**	**34 (40)**	**60 (26)**	34 (20)	**47 (21)**	66 (21)	62 (42)	63 (19)	**36 (11)**	42 (12)
**Age**											
<50 years	52	84 (19)	56 (42)	74 (26)	43 (27)	59 (21)	69 (24)	79 (33)	68 (19)	44 (12)	44 (12)
≥50 years	44	**58 (25)**	**39 (40)**	**59 (25)**	35 (21)	51 (22)	70 (20)	**55 (43)**	64 (21)	**34 (11)**	43 (13)
**Duration of disease**											
≤8 years	47	73 (28)	49 (42)	69 (28)	42 (23)	55 (20)	68 (24)	72 (38)	63 (20)	41 (13)	43 (13)
>8 years	46	71 (23)	44 (42)	65 (26)	37 (27)	56 (24)	69 (21)	64 (42)	68 (20)	39 (12)	44 (12)
**Co-morbidities**											
CLD	62	70 (25)	41 (41)	63 (26)	37 (23)	54 (23)	65 (23)	60 (41)	63 (21)	38 (12)	41 (13)
Sinusitis	48	73 (25)	44 (43)	66 (28)	36 (23)	54 (22)	68 (22)	65 (42)	65 (22)	39 (12)	42 (13)
Diarrhea	42	65 (26)	37 (40)	60 (29)	34 (24)	51 (23)	65 (24)	54 (41)	63 (22)	36 (12)	41 (13)

*SF-36 scales: PF, physical functioning; RP, role-physical; BP, bodily pain; GH, general health; VT, vitality; SF, social functioning; RE, role-emotional; MH, mental health; PCS, physical component summary; MCS, mental component summary; CLD, chronic lung disease; CVID, common variable immunodeficiency*.

*Bold – *p* < 0.05*.

*Totals may vary because of missing values*.

**Table 2:** **Mean values of SF-36 scales for CVID patients compared with healthy subjects and with patients with different chronic diseases in Italy**.

	*N*	PF	RP	BP	GH	VT	SF	RE	MH
Healthy subjects	608	97.3	94.3	89.2	80.2	72.2	86.4	88.0	75.8
Diabetes	98	62.9	59.7	59.8	43.6	47.6	66.9	57.6	53.4
Heart failure	129	49.5	43.1	47.6	35.4	38.3	54.8	46.3	46.6
Cancer	34	60.6	62.4	57.0	44.9	48.3	64.1	58.6	49.9
Chronic obstructive pulmonary disease	188	58.7	49.5	52.0	41.5	45.8	62.6	55.8	53.5
Mental disorders	180	65.4	49.5	51.9	44.7	40.9	55.0	43.7	42.0
CVID	96	72.4	47.3	67.2	39.3	55.2	69.5	68.3	66.3

*SF-36 Scales: Physical Functioning (*PF)*, Role-Physical (*RP)*, Bodily Pain (*BP)*, General Health (*GH)*, Vitality (*VT)*, Social Functioning (*SF)*, Role-Emotional (*RE)*, Mental Health (*MH)*. SF-36 values for Italian diseases (12)*.

#### Longitudinal variation of SF-36

**Figure 2:**
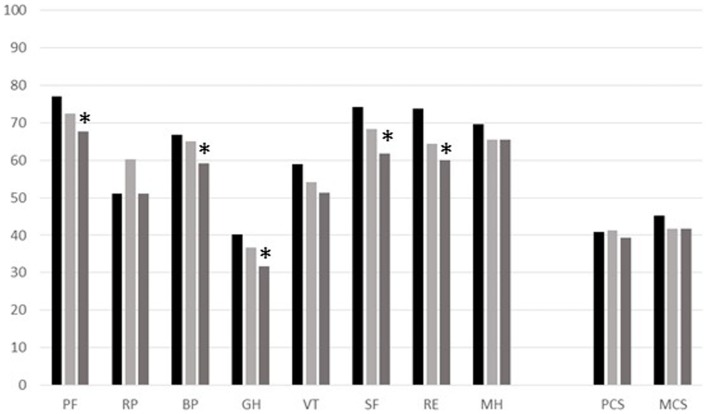
**Profile of the mean values for each scale of SF-36 for the group of 66 CVID patients observed at different times (T0, black column; T1, pale-gray column; T2, gray column)**. SF-36 Scales: Physical Functioning (PF), Role-Physical (RP), Bodily Pain (BP), General Health (GH), Vitality (VT), Social Functioning (SF), Role-Emotional (RE), Mental Health (MH), Physical component summary (PCS), and Mental component summary (MCS). *Significant *p-*values between T0 and T2.

Longitudinal variations in SF-36 scores were observed over the whole sample, as well as over the subsample of 66 patients attending all three sequential assessments. The scores observed considering the SF-36 mean values in the 66 patients, who were followed at all three points of observation times, are shown in Figure 2. Differences in the scores of SF-36 scales between T0 and T2 are statistically significant for the following scales: PF (*p* = 0.03), BP (*p* = 0.05), GH (*p* = 0.02), SF (*p* = 0.002), and RE (*p* = 0.03). However, we should consider the age dependence of all scales, especially because patients at T2 were 6 years older than at T0. Moreover, we found no differences in HRQoL scales between patients on replacement with IVIG and SCIG.

#### GHQ assessment and PtGA/PhGA

GHQ-12 assessment showed that more than 35% of the patients were at risk of anxiety and depression (GHQ-positive) at all observation times (Table 3). This percentage was increased to about 70% in females. The disease severity perception graded by patients and by physicians at the three points of observation is reported in Table 3. Differences between the perception of patients and physicians were more evident at T2, with higher percentage of low severity grade reported by physicians. As expected, PhGA was greater in GHQ-positive patients. Twenty-five percent of GHQ-positive patients considered their disease severity as high.

**Table 3:** **Gender, GHQ status, and disease severity reported by patients and by physicians at each assessment (numbers and percentages)**.

	All	*n*.96 (T0) (%)	*n*.92 (T1) (%)	*n*.66 (T2) (%)
Sex	Males	52	51	52
	Females	48	49	48
GHQ+		36	39	37
GHQ+	Males	28	23	33
	Females	72	77	67
PtGA	Low	27	17	38
	Moderate	49	49	40
	High	24	33	22
PhGA	Low	17	16	47
	Moderate	50	51	39
	High	33	33	14

*PtGA, Patient Global Assessment; PhGA, Physician Global Assessment*.

*GHQ, General Health Questionnaire; GHQ+, GHQ-positive/“cases” or GHQ ≥4*.

The 15 patients who were permanently GHQ-positive at all observations had constantly low mean values of scores on SF-36 scales. Patients with a highly severe perception of the disease (PtGA) and patients who were judged as seriously affected by the physicians (PhGA) reported a lower health status with respect to the others.

### General Health Questionnaire data and variations of mean values in specific SF-36 scales

To analyze the relationship between SF-36 and GHQ-12 data, variation in GHQ status between T0 and T2 were coded as 1 when GHQ passed from 0 to 1 (GHQ-worsened) and as 0 in all other instances (GHQ-stable/improved). Changes in SF-36 scales were recorded as absolute differences between values at T0 at T2. Average variation in SF-36 scores was then compared between the group of GHQ-worsened and GHQ-stable/improved. The SF-36 scales showing variations significantly different in the two groups (GHQ-worsened and GHQ-stable/improved) were selected.

Over the 6-year observational period, the general health condition of 11/66 patients worsened, passing from “GHQ-negative” to “GHQ-positive” status, i.e., showing symptoms of psychological distress.

**Figure 3:**
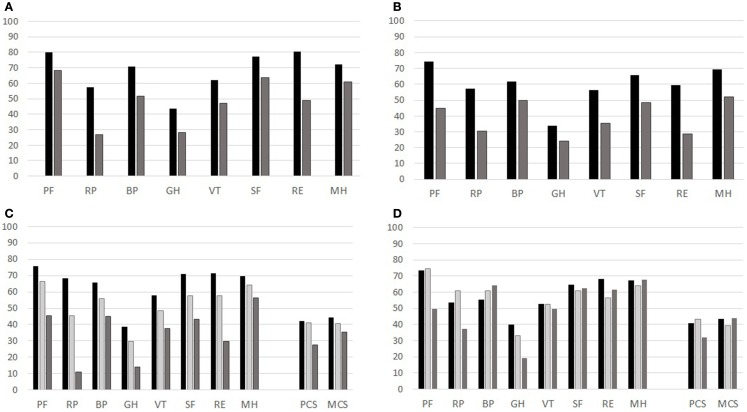
**Mean values for each scale of SF-36 for the group of 66 CVID patients observed at different times (T0, T1, T2), GHQ status (T0 and T2) and PtGA and PhGA assessments**. **(A)** GHQ at T0: black column GHQ-negative; gray column: GHQ-positive. **(B)** GHQ at T2: black column GHQ-negative; gray column: GHQ-positive. **(C)** PtGA (black column: very mild/mild; pale-gray column: moderate; gray column: severe/very severe). **(D)** PhGA (black column: very mild/mild; pale-gray column: moderate; gray column: severe/very severe).

These patients also registered a negative score variation on SF-36 scales: Physical Functioning, Vitality, Social Functioning, and Mental Health. In a logistic regression, controlled for age and gender, we noticed that a decrement of 1 point in each of the four mentioned scales increases the risk of developing anxiety/depression from 3 to 5% (Table 4). Thus, the GHQ-12 and SF-36 deteriorations were strictly linked. To convey an idea of the magnitude of the effect, we considered the cumulative observed variation of the Physical Functioning scale (PF) over the follow-up period: 10 points. The odds ratio of 1.05 means that a patient affected by the average negative variation of PF has an increased risk of 5% per year of psychological distress. Also VT, SF, and MH subscales were strongly influenced by GHQ status (Figure 3).

**Table 4:** **Odd ratio (OR) and *p-*values for SF-36 scales for “changes” in GHQ-12 status (from negative to positive)**.

SF-36 scale	OR	*p-*value
PF	1.05 (1.01–1.06)	0.012
VT	1.04 (1.01–1.05)	0.008
SF	1.03 (1.01–1.06)	0.024
MH	1.04 (1.01–1.07)	0.03

### Immunological data and HRQoL

In CVID, we have previously identified (15) a severe clinical phenotype, characterized by low IgA level (<7 mg/dL) and low switched memory B cells, confirming previous observations showing that the loss of function of memory B cells seems to represent the major cause of CVID-associated clinical conditions (16). Moreover, clinical improvement of CVID was observed in patients receiving high Ig dosages, >600 mg/kg/months (17, 18). These dosages might allow to keep IgG trough levels >600–800 mg/dL. We then grouped our CVID cohort on the basis of three defined parameters: IgA >7 mg/dL, IgG trough levels >600 mg/dL, switched memory >2%. At T0, 67% of the patients had IgA levels <7; 50% had switched memory B cells <2%; 61% had IgG levels >600 mg/dL (a percentage ranging from 14 to 26 has missing information on these parameters).

The analysis of the mean values on SF-36 scales in patients with or without each defined parameter showed no statistically significant differences among groups.

### Low HRQoL was predictive of mortality

Patients’ mortality during the follow-up was registered with exact date of death. According to this outcome, patients at T0 were divided into two groups: survivors vs. deceased. Cox Survival analysis was performed, introducing as covariates the SF-36 scales that showed marked differences between survivors and deceased at T0. Age and gender were also included in the analysis in order to estimate the role of HRQoL in predicting mortality, as adjusted by these two variables. For the SF-36 scales independently predictive of mortality, we operated a dichotomization of values, indicating “at-risk” and “not at-risk” patients. The cut-off values were selected observationally based on points of disruption in the trend of number of death over SF-36 scores. For SF-36 scales predictive of mortality, we graphed the two groups (“at-risk” and “not at-risk”) with Kaplan–Meier survival curves, whose significance was verified by Log-rank test. A new Cox survival analysis with SF-36 scales dichotomized was produced, in order to assess the specific risk of death for the patients that scored under the cut-off at T0. The most evident difference between the two groups (Table 5) is, not surprisingly, in terms of age: survivors are significantly younger than deceased patients. However, score values of Physical and Social Functioning as well as Role-Emotional at T0 seem to be remarkably reduced for patients that died during the study time. This result was partially confirmed when adjusted by age: except for the Role-Emotional, which has no age-independent effect on mortality, both Physical and Social Functioning score values maintain their significant predictive power.

**Table 5:** **Characteristics of survival and deceased patients**.

Characteristics	All patients, *N* = 96	Survived patients, *N* = 78	Deceased patients, *N* = 18	*p-*Value
**SF-36 scores, mean (SD)**				
PCS	39.8 (12.4)	40.8 (12.5)	35.5 (10.9)	0.06
MCS	43.4 (12.2)	44.0 (12.1)	41.0 (12.9)	0.18
Physical Functioning	72.4 (25.4)	76.9 (23.4)	53.8 (25.8)	0.001
Role-Physical	47.3 (42.3)	50.3 (42.4)	34.7 (40.3)	0.08
Body Pain	67.1 (26.5)	67.5 (26.9)	65.9 (25.9)	0.41
General Health	39.3 (24.5)	40.1 (25.3)	36.0 (21.2)	0.27
Vitality	55.2 (21.8)	56.8 (21.1)	48.6 (23.8)	0.08
Social Functioning	69.5 (22.2)	71.5 (21.6)	61.1 (23.4)	0.04
Role-Emotional	68.3 (39.8)	73.1 (37.3)	48.1 (44.6)	0.008
Mental Health	66.3 (19.9)	67.6 (19.1)	60.7 (22.6)	0.09
Age, mean years (SD)	48.2 (17.0)	44.8 (15.7)	62.9 (14.7)	0.0001
Gender, men%	52.2	52.6	50.0	0.84
GHQ, cases%	26.6	28.6	27.7	0.36
IgA, >7 mg/dL%	32.9	33.3	30.0	0.83
IgG, >600 mg/dL%	61.0	63.9	40.0	0.5
SW mem, >2%	50.0	50.8	44.4	0.72

**Figure 4:**
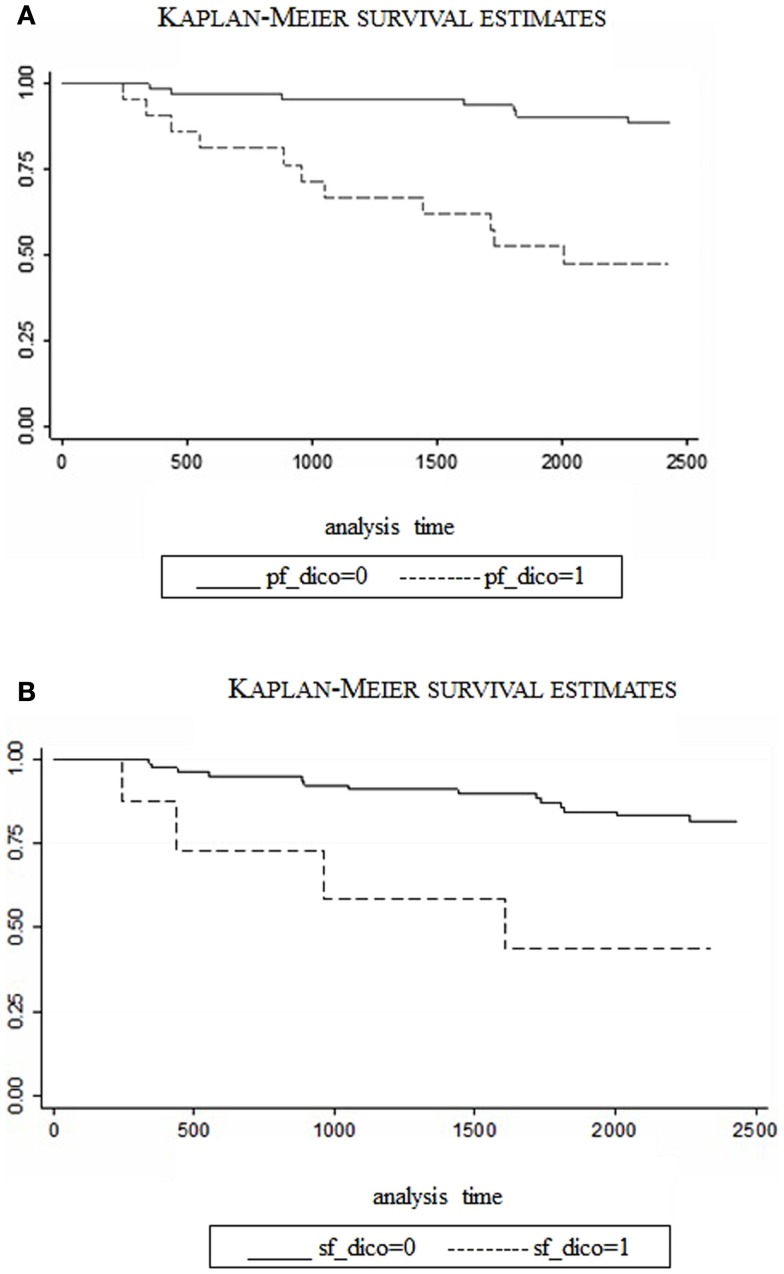
**Survival rates for CVID patients (“at-risk” vs. “not at-risk”) considering SF-36 scales: Physical Functioning, cut-off at 50 (A) and Social Functioning cut-off at 37.5 (B)**. (dico: dichotomized).

The relative risk (RR) of death associated with PF and SF scales is 0.98 and 0.97, respectively, meaning that each point increase in Physical and Social Functioning scores, independently of age, reduced the risk of death by 2% and 3%. The two predictive scales (PF and SF) were then dichotomized based on the observed point of disruption in the trend of number of deaths per different scores of PF and SF. More specifically, a cut-off value of 50 was selected for PF and a value of 37.5 for SF. People with values higher than the cut-off were classified as “not at-risk,” compared to the “at-risk” below the cut-off. In a survival analysis with these dichotomized variables, PF scores <50 were associated with a RR of 4.4 (CI: 1.7–11.8, *p* < 0.03) and SF scores <37.5 determined a RR of 10.0 (CI: 2.6–37.9, *p* < 0.001). In other words, all other variables being equal, patients with scores below the cut-off in Physical Functioning have 4.4 times the risk of dying than patients with higher scores; the same risk is 10 times higher for patients under the cut-off in social functioning. Figures 4A,B compare survival rates over the follow-up period, between “at-risk” and “not at-risk” patients as classified by T0 for PF/SF scores. The difference is extremely significant with the “at-risk” group survival curve always below the “not at-risk” curve (Log-rank test <0.0001). The median value was approached in both the “at-risk” groups, meaning that half of the patients have died at 48 months (SF) and 60 months (PF), whereas, the percentage of death in the “not at-risk” groups is lower than 25% at the end of the observation period (72 months).

## Discussion

Patient reported outcome measures in clinical practice, in particular, those evaluating HRQoL (19), have been proposed as a means of facilitating doctor–patient communication, uncovering patients’ problems, as well as monitoring disease or treatment, and as a screening for functional problems (20, 21). In a previous study on HRQoL performed in our cohort of CVID patients, we showed (7) a low HRQoL in particular in physical domains: the Role-Physical and the General Health scales of the SF-36 questionnaire. Moreover, we showed that being female, older, and GHQ-positive proved to be major risk factors associated with a poor health status. Here, we extended the study time over a 6-year period. To our knowledge, this is the first longitudinal assessment of HRQoL in adult CVID patients. We confirmed that HRQoL was lower than that reported in generally healthy population with mental health scales less affected than physical scales. Moreover, Physical Role and General Health scales showed the worse scores at all observational times and were lower than those reported in patients with other chronic disease entities (12), with the exception of patients affected by heart failures. Moreover, we showed that about one-third of patients were at risk of anxiety/depression at all observation times, a percentage that reached two-thirds of patients, considering only the group of females. The crucial role of anxiety/depression symptoms on HRQoL has been proven recently also in a pediatric population affected by primary immunodeficiencies, where the disease was less likely to affect physical functioning than psychosocial functioning (22). Patients tested continuously that GHQ-positive had a perception of the disease as severe and recorded low mean values of all SF-36 scales. Thus, the GHQ-12 and SF-36 deteriorations were strictly linked. However, it is impossible to verify which might be the *primum movens*. Since patients with a decrease of the Physical Functioning, the worse SF-36 scale in CVID, increased their risk of psychological distress about 50% during the observational time, it is possible to hypothesize that the disease might be the major cause of depression/anxiety. Our data on HRQoL confirmed the data reported 10 years ago in the first multidimensional assessment on HRQoL in CVID (23) and more recently in a survey run by IPOPI (4). However, SF-36 scales showed less severe defects than those previously reported, possibly because of the over-representation of females in both studies. All studies recognized limitations in work and other daily activities as a result of declined physical health and general health. HRQoL measures ensure that treatment and evaluations are focused on the patient rather than on the disease, and may be used as a way of capturing the personal and social context of patients and linking it to the classical clinical view of the disease. However, while HRQoL measures are now quite commonly included in the protocols of randomized, controlled clinical trials and other clinical studies, their use in routine clinical practice is still quite limited; they were never used in combination with other questionnaires, limiting the possibility to identify correct measures of intervention. In fact, our experience in the evaluation of the HRQoL in a day hospital setting for CVID patients, confirmed what has been observed in other diseases, namely that GHQ-positive patients (with minor psychiatric, non-psychotic diseases such as depression or anxiety) suffer a lot (13). Thus, in the comprehensive evaluation of the patients’ status, their psychological condition and disorders such as anxiety/depression should be evaluated in a global HRQoL assessment as they yet often go unrecognized. Counseling can be useful in GHQ-positive patients, since an improvement of GHQ status might lead to a better HRQoL. On the other hand, an improvement in HRQoL can be associated with improvement in psychological well-being. Our results might be helpful in the interpretation of data currently available on quality of life in CVID patients receiving different immunoglobulin treatment options. A recent study from IPOPI (4) reported that most patients were satisfied with their current therapy. Significant differences in satisfaction were seen when comparing SCIG with IVIG administration; SCIG respondents were more satisfied with treatment than IVIG respondents. Since we have shown that the perception of disease severity was linked to the GHQ status and that GHQ-positive patients will perceive their disease as more severe, without a parallel assessment of the psychological status of patients receiving SCIG or IVIG, it might be difficult to attribute an advantage to a treatment modality vs. another. Moreover, as the authors claimed, the results of the survey done on patients affiliated to the IPOPI, might not represent all people with primary immune deficiencies treated with immunoglobulin therapy. Despite this limitation, their data indicated that the primary immunodeficiency impacted on quality of life, also taking into account the favorable effect of immunoglobulin treatment. In our study, the clinical condition, as opposed to the different treatment strategies with immunoglobulins, had a major role on the deterioration of HRQoL. Values of Physical and Social Functioning as well as Role-Emotional scales of SF-36 at T0 were remarkably reduced for patients that died during the study time. Even if survivors were significantly younger than deceased patients, with the exception of the Role-Emotional, which has no age-independent effect on mortality, both Physical and Social Functioning maintained their significant predictive power. The RR of death for PF and SF was 0.98 and 0.97, respectively, meaning that each point increase in PF and SF, independently of age reduces the risk of death by 2 and 3%, respectively. We have already shown (24) that malignancies are the major cause of death in patients with adult onset CVID: the high rate of mortality (about 20% in a 40-year follow-up) was similar to that reported in a study run in the United States of America over a similar period of follow-up (25). The high rate of mortality observed here further confirmed the need to focus our attention on early diagnosis of non-lymphoid and lymphoid cancers that influence lives and HRQoL in CVID. Monitoring health status in long-term longitudinal studies with generic tools, such as SF-36 and GHQ-12, may provide information about clinical status, therapy efficacy, and eventually a modification in physical or mental status. It is relevant to consider here the specificities of each area of the SF-36 health status and the GHQ-12 assessments, taking into consideration any effort to reduce the physical burden of the disease. However, a major limitation in the assessment of HRQoL in CVID is still the absence of a disease-specific questionnaire available to monitor quality of life in many other diseases. We are now developing and validating a CVID-disease-specific questionnaire on HRQoL necessary to complement evidence-based guidelines and policies.

## Author Contributions

Stefano Tabolli: designed research, analyzed data, and wrote the manuscript; Patrizia Giannantoni: performed the statistical analysis; Federica Pulvirenti: performed research, collected, and analyzed data; Fabiola La Marra: performed research, collected, and analyzed data; Guido Granata: performed research, collected, and analyzed data; Cinzia Milito: performed research, collected, and analyzed data; Isabella Quinti: designed research, analyzed data, and wrote the manuscript.

## Conflict of Interest Statement

The authors declare that the research was conducted in the absence of any commercial or financial relationships that could be construed as a potential conflict of interest.
